# Promoting water safety for children in Iran

**Published:** 2023-12

**Authors:** Ali Davoudi-Kiakalayeh

## Abstract

Unfortunately, more than 1250 people are drowning in Iran every year as a result of unintentional drowning. Eighty-five percent were men and 13% of the cases involved were children under the age of 15. Public awareness campaigns can be an effective prevention strategy when combined with other ongoing prevention efforts. A public awareness campaign called The Darya Program was developed to raise awareness in northern Iran about water safety at beaches, inland rivers, lakes and dams, and general water safety. This campaign was aired on television during the peak summer bathing season in northern Iran. The campaign aimed to raise awareness of water safety issues and appropriate safety precautions among the general community (1-3). The key messages of The Darya Program for children and their parents included:

- Always supervise children around water

- Never swim alone

-Swim only in the protected area of beaches

-Be aware of fast-moving water, submerged objects and deep water

-Teach your children to swim, it's great!

-Learn CPR

-Life jackets should be used by children for all activities while in and around natural waters

Preventing childhood drowning involves a combination of strategies, including education, physical barriers, adult supervision, and swim lessons. Education is a critical component of drowning prevention. It includes teaching children about the potential risks associated with swimming, especially for children with epilepsy or other medical conditions. It is also important to educate adults about safe rescue and resuscitation techniques.

Physical barriers such as pool fences, pool covers, and water entry alarms can significantly reduce the risk of drowning. Pool fencing is especially effective for young children. It should completely surround the pool and isolate it from the house. Legislation should require isolation fencing with secure, self-locking gates for all pools; public, semi-public and private ones.

Adult supervision is essential to prevent drowning. Children should always be supervised in and around water. This supervision should be continuous, especially for infants and other children who lack mobility. The age at which children need such supervision has not been established but is likely to be at least five years.

Swimming education is another effective preventive measure. Children should learn basic swimming and water safety skills. This includes learning how to swim and how to save themselves and others in an emergency situation. Formal swimming lessons may be particularly beneficial.

The use of personal flotation devices, such as life jackets, can also help prevent drowning. These devices can keep a child afloat and buoyant in the water, increasing their chances of survival.

The promotion of immediate resuscitation before the arrival of medical personnel should be encouraged everywhere. Such resuscitation significantly increases the likelihood of a good outcome - regardless of age, gender, duration of immersion, or the presence of hypothermia.

In summary, drowning prevention involves a combination of education, physical barriers, adult supervision, swim training, use of personal flotation devices, and immediate resuscitation. These measures should be implemented in a manner appropriate to the child's developmental level and cultural acceptability.

